# Iron Fortification and Inulin Supplementation in Early Infancy: Evaluating the Impact on Gut Microbiome in a Piglet Model

**DOI:** 10.1016/j.cdnut.2025.104587

**Published:** 2025-03-15

**Authors:** Jungjae Park, Cynthia Jinno, Saumya Wickramasinghe, David A Mills, Yanhong Liu, Bo L Lönnerdal, Peng Ji

**Affiliations:** 1 Department of Nutrition, University of California, Davis, CA, United States; 2 Department of Animal Science, University of California, Davis, CA, United States; 3 Department of Food Science and Technology, University of California, Davis, CA, United States

**Keywords:** inulin supplementation, iron fortification, gut microbiome, infant formula, pig model

## Abstract

**Background:**

Prophylactic iron fortification in infant formula effectively prevents iron deficiency anemia. However, the low absorption rate results in excess unabsorbed iron accumulates in colon, where it has been linked to harmful microbiota changes and increased diarrheal incidence. Prebiotic oligosaccharides have shown promise in mitigating these adverse effects, but the role of inulin or synbiotic supplementation with inulin-fermenting lactic acid bacteria in modulating early gut microbiome under iron fortification remains understudied.

**Objectives:**

This study used a neonatal pig model to investigate the effects of iron fortification and inulin supplementation, with or without *Ligilactobacillus agilis* YZ050 (*L. agilis*), on gut microbiome.

**Methods:**

Twenty-four piglets were stratified and randomly assigned into 1 of the 4 dietary treatments from postnatal day (PD) 2: iron-adequate milk (AI), high-iron milk (HI), high-iron milk with 5% inulin (HIP), or HIP milk with oral gavage of *L.agilis* every third day (HIS). Piglets were individually housed and fed milk in proportion to body weight in 14 meals daily, simulating formula feeding in infants. Fecal and colonic microbiome were analyzed via 16S rRNA sequencing, with microbial diversity and relative abundance analyzed using QIIME2 and R.

**Results:**

Iron fortification, regardless of inulin supplementation, decreased α-diversity compared with AI. β-Diversity showed clustering of HIP and HIS samples, which were distinct from AI and HI. Although iron fortification had minor impact on microbial composition, inulin supplementation significantly modified microbiome diversity, increasing *Prevotella*, *Megasphaera*, and *Lachnospiraceae*_NK3A20_group species, while reducing *Bacteroides* and *Ruminococcus*. Colonic microbiome shifted from *Bacteroides*-dominant enterotype in AI and HI groups to *Prevotella*-dominant enterotype in HIP and HIS groups, indicating enhanced fiber degrading capacity. Despite its inulin-fermenting property, *L.agilis* showed limited colonization and minimal microbiome impact.

**Conclusions:**

Inulin supplementation significantly influenced gut microbiome, shifting enterotype from *Bacteroides* to *Prevotella*. dominance and overriding the effect of high-iron fortification in a milk-fed piglet model.

## Introduction

Differences in nutrient composition between human milk and cow milk–based infant formula contribute to distinct gut microbiome profiles, with breastfed infants harboring a greater abundance of beneficial commensals. Iron content markedly differs between human milk and infant formula. Due to low-iron content in cow milk, most infant formulas in the United States are fortified with 12 mg Fe/L to prevent iron deficiency anemia (IDA) in infancy [1,2]. Given the low absorption rate of fortified iron (<10%), a substantial amount of unabsorbed iron reaches the colon and influence gut microbiome [[3], [4], [5]]. Notably, beneficial bacteria, such as *Lactobacillus* species, thrive in iron-restricted environments [6,7], and iron fortification/supplementation has been shown to reduce its abundance in both animal and human studies [8,9]. For example, a double-blind randomized controlled trial (RCT) in Kenyan infants found that iron supplementation through micronutrient powder (MNP) increased enteric pathogens, such as *Salmonella* sp., *Clostridium difficile*, *Clostridium perfringens*, and pathogenic *Escherichia coli*, while reducing *Lactobacillus* and *Bifidobacterium* species [10]. Similarly, an RCT in Ivorian children showed that iron fortification (20 mg Fe/d) in biscuits significantly reduced fecal lactobacilli and increased enterobacteria, which include potential pathogens [9]. Although iron prophylaxis is critical for preventing IDA in infants and young children, it is equally important to develop dietary strategies to mitigate its potential adverse effects on the gut microbiota.

Beyond iron content, human milk contains a diverse group of oligosaccharides (5–15 g/L), which are either absent or present at much lower concentrations in cow milk [11]. Human milk oligosaccharides (HMOs) function as natural prebiotics, selectively promoting beneficial bacteria, primarily *Bifidobacterium* and certain *Lactobacillus* species, in the infant gut [12]. Additionally, HMO-using microbes can crossfeed other bacterial species, thereby enhancing gut microbial diversity. Despite the presence of cow milk oligosaccharides, the deficiency in primary HMOs in cow milk–based infant formula contributes to the distinct microbiome composition observed in formula-fed infants.

Numerous studies have examined the effects of prebiotic supplementation in formula-fed infants, particularly focusing on galacto-oligosaccharides (GOSs) and fructo-oligosaccharides [11,13,14]. For instance, supplementing GOS alongside iron-fortified MNP was found to alleviate the negative effects of iron fortification on the intestinal microbiota of Kenyan infants [15]. Inulin, a nondigestible β(2-1) fructan with polymerization degree of 2–60, also exhibits prebiotic properties by stimulating *Bifidobacterium* sp. [[16], [17], [18]] and has mild laxative effects [[19], [20], [21]]. Moreover, inulin supplementation has been shown to enhance iron bioavailability in 6-wk-old weaned pigs under conditions of low-iron intake [22,23]. Interestingly, our recent study found that 5% inulin supplementation not only attenuated body iron overload but also reduced colonic iron concentration in piglets fed iron-fortified milk replacer [5]. However, few studies have explored the combined effects of inulin supplementation and iron fortification on gut microbiome of formula-fed infants.

In this study, we used milk-fed piglets as a preclinical model for formula-fed infants to investigate the effects of iron fortification and inulin supplementation on gut microbiota. Given the sensitivity of *Lactobacillus* sp. to iron fortification and the potential implications for gut health, probiotic supplementation has been proposed as a strategy to support beneficial bacteria populations, particularly during iron supplementation in early infancy [9]. Therefore, we further examined the synbiotic effect of supplementing inulin with *Ligilactobacillus agilis* YZ050, an inulin-fermenting lactic acid bacterium [24], on gut microbiome under iron fortification.

## Methods

### Study design, animal management, and sample collection

The study protocol was approved by the Institutional Animal Care and Use Committee at the University of California, Davis (UCD). The details of study design, animal management, and sample collection have been previously published [5]. Briefly, 24 neonatal piglets (equal number of females and males) were stratified by birth body weight (BW) and sex, then randomly assigned to 1 of the 4 dietary treatments (*n* = 6/treatment) at postnatal day (PD)2. From PD2 to PD29, piglets received an iron-adequate cow milk replacer [AI group; 60 mg iron/(kg milk solids) or 12.4 mg iron/L milk] supplemented with 5% (wt/wt milk solid) maltodextrin, a high-iron milk replacer [HI group; 480 mg iron/(kg milk solids) or 98.9 mg iron/L milk] supplemented with 5% maltodextrin, the HI milk supplemented with 5% inulin (Orafti IPS; Beneo) as prebiotics (HIP group), or the HIP milk and an oral gavage of *L agilis* YZ050 (2.5 × 10^9^ CFU/mL in 5-mL de Man, Rogosa, and Sharpe medium) once every 3 days during the study (HIS group). As the strain displays robust activity in degrading inulin and releasing fructose [24,25], combined supplementation of inulin and this potentially probiotic strain is expected to act synergistically as a synbiotic. Vehicle control (de Man, Rogosa, and Sharpe medium) was not applied to piglets in the other treatments in this study. The iron supplementation concentrations were carefully selected based on the National Research Council (2012) nutrient requirement guidelines for swine. The guideline indicates that milk-fed piglets require 50–150 mg iron per kg of milk solids to prevent IDA and support healthy growth. The AI milk formulation meets these requirements, whereas the iron concentration in the HI milk is 3 times the highest recommended value, mimicking excessive iron fortification in infant formula. Piglets were weighed every other day and fed 270 mL milk/(kg BW/d) according to the most recent BW. Fecal samples were obtained using a rectal swab on PD2 (baseline), PD8, PD15, PD22, and PD29. All piglets were killed on PD29, and proximal colon contents were collected. The samples were snap-frozen in liquid nitrogen and then stored in −80°C until further analysis.

### Microbial DNA Extraction, library preparation and 16S rRNA sequencing

Bacterial DNA was extracted from feces and proximal colon contents (∼100 mg) using the Quick-DNA Fecal/Soil Microbe Kit (Zymo Research, Irvine, CA) following the manufacturer’s instructions.

The microbiome library preparation was done following a procedure previously described [26]. The 16S rRNA genes of bacterial DNA were amplified by PCR using a primer set that targets the V4 region (515 F: 5'-XXXXXXXXGTGTGCCAGCMGCCGCGG TAA-3'; 806 R: 5'-GGACTACHVGGGTWTCTAAT-3') and contains an 8-bp barcode (X) and Illumina adapter (GT). Each 25-μL PCR reaction contained 2 μL DNA template, 9.5 μL nuclease-free water, 12.5 μL GoTaq 2× Master Mix (Promega), and 0.5 μL of each V4 forward and reverse primer (10 μM). The PCR was performed with the following protocol: *1*) 94°C for 3 min for denaturation, *2*) 94°C for 45 s, *3*) 50°C for 1 min, *4*) 72°C for 1.5 min (steps 2–4 were repeated for 35 times), and *5*) 72°C for 10 min for elongation. Each sample was amplified via PCR in triplicates. Ultrapure Milli-Q water and DNA elution buffer from the kit were incorporated as negative controls in the analysis to evaluate potential reagent and environmental contaminations. The DNA library was prepared by pooling PCR products of all bacterial DNA samples extracted from colon digesta and feces of piglets. The volume of each sample added to the library was subjectively adjusted based on (Invitrogen) band intensity stained with SYBR safe in a 2% agarose gel. The pooled sample was purified using the QIAquick PCR Purification Kit (Qiagen) and then submitted to the UCD Genome Center for 250-bp paired-end sequencing on the Illumina MiSeq platform.

### Microbiome data processing, taxonomic classification, and visualization

The microbiome analysis was performed following a procedure previously described [27,28]. Data processing and statistical analysis were conducted using the Farm Cluster, a Linux-based supercomputing cluster for the College of Agricultural and Environmental Sciences at UCD, and R statistics software, respectively. Sabre (https://github.com/najoshi/sabre) was used for demultiplexing and trimming sequence reads by aligning sequences to its sample of origin based on the barcode and removing barcodes from each end of raw sequences. Reads not matching any barcode and sequences or failing to meet the minimum quality thresholds were discarded. The sequences were then imported into Quantitative Insights Into Microbial Ecology 2 (QIIME2; version 2022.2.0) for downstream filtering and bioinformatics analysis [29]. To optimize data quality, the DADA2 plugin was used to truncate reads at 240 nucleotides—selected after testing various lengths for error rates—remove primers, denoize paired-end reads, and exclude any reads not matching a barcode or meeting predefined quality thresholds [30]. Taxonomic classification was assigned using the taxonomy-classifier plugin, the SILVA rRNA V4 database (version 138.1) [31]. The amplicon sequence variant table and phylogenetic tree were imported into R (version 4.2.3) using the phyloseq package for analysis of alpha-diversity, beta-diversity, relative abundance. The key taxon features characterizing the microbiome composition of the treatments were analyzed for differential abundance using the DESeq2 package. The 16S rRNA sequencing data have been deposited in the NCBI Sequence Read Archive database under BioProject ID PRJNA1236018. The project information is accessible at the following link: http://www.ncbi.nlm.nih.gov/bioproject/1236018.

### Statistical analysis

Statistical analyses were performed using the phyloseq package in R (version 4.2.3), with data visualization conducted using the ggplot2 package in R and QIIME2 [32]. Normality was assessed with the Shapiro–Wilk test, and homoscedasticity was checked using Bartlett test for α-diversity and relative abundance data. The fecal microbiome analysis used a statistical model that included treatment, day, and their interaction, whereas the colon microbiome analysis only included treatment as the main effect. α-Diversity and relative abundance of bacterial taxa were analyzed using the nonparametric Kruskal–Wallis rank sum test, followed by Dunn post-hoc test or Conover test for multiple comparisons, implemented via the agricolae package in R. For β-diversity analysis, Bray–Curtis dissimilarity was used in principal coordinates analysis (PCoA). Statistical significance of β-diversity was determined through PERMANOVA with 999 permutations using the vegan package. *P* values for relative abundance were adjusted for multiple testing using the Benjamini–Hochberg correction to control the false discovery rate. Statistical significance was declared at *P*-adjusted value of <0.05, with a trend toward significance considered at *P*-adjusted value of <0.1.

## Results

### Diversity of colonic and fecal microbiome on PD29

The 16S rRNA sequencing yielded an mean of 37,310 reads per sample, which were reduced to 10,869 per sample after quality filtering, removal of reads mapped nontarget organisms—including Archaea, chloroplasts, and the phylum Cyanobacteria—and rarefaction. The phyloseq object removed sequences that did not map to known taxa within the SILVA database. A minimum prevalence threshold was applied, retaining only taxa that accounted for ≥1.0% of the total microbial composition in each sample. The final phyloseq object for colonic and PD29 fecal samples contained 1025 taxa.

α-Diversity analysis revealed that high-iron diets (HI, HIP, and HIS) significantly reduced Chao1 index (*P* ≤ 0.02) and tended to reduce Shannon index (*P* ≤ 0.06) in colon compared with the AI, with no differences among HI, HIP, and HIS groups (Table 1). Neither Chao1 nor Shannon index of fecal microbiome was affected by treatment (Table 1). For the HIP group, the Chao1 index of colonic microbiome was significantly lower than that in fecal microbiome (*P* < 0.05), whereas no significant differences in α-diversity indices was observed between colon and feces within other treatments.

**TABLE 1:** α-Diversity of colonic and fecal microbiomes on postnatal day 29.

Index	Site	AI	HI	HIP	HIS
Chao1	Colon	253.1 ± 13.8^a^	205.5 ± 13.9^b^	188.3 ± 7.9^b,^^1^	183.5 ± 13.3^b^
	Feces	247.8 ± 7.6	238.7 ± 10.6	233.8 ± 12.6	222.3 ± 12.2
Shannon	Colon	4.0 ± 0.1	3.7 ± 0.2	3.6 ± 0.1	3.6 ± 0.1
	Feces	4.0 ± 0.1	3.8 ± 0.1	3.9 ± 0.1	3.8 ± 0.1

Values are presented as mean ± SE.

Abbreviations: AI, iron-adequate milk diet; HI, high-iron milk diet; HIP, high-iron milk diet with prebiotic; HIS, high-iron milk diet with symbiotic.

^a,b^Within the site, values that share no common letter differed significantly across treatments.

1 Within the treatment, values differed significantly between sites.

**FIGURE 1:**
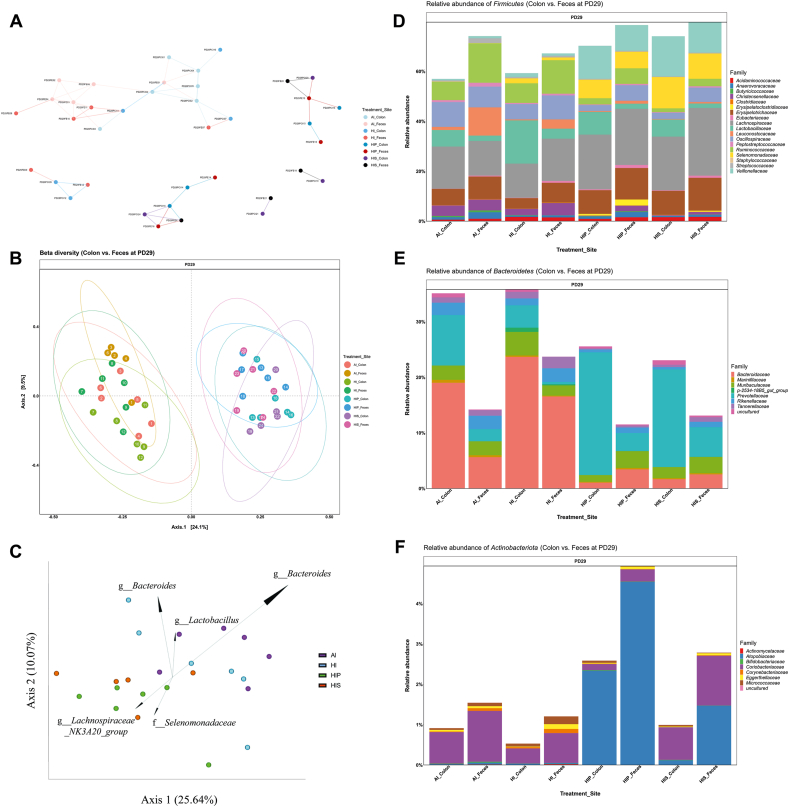
β-Diversity and relative abundance of colonic and fecal microbiome on postnatal day (PD)29. The β-diversity of colonic and fecal microbiome on PD29 presented as Jaccard network plot (A) and Bray–Curtis distance in a principal coordinates analysis (PCoA) plot (B). PCoA biplot (C) highlighted key genera or family (Selenomonadaceae) contributing to sample clustering. Relative abundance of Firmicutes (D), Bacteroidetes (E), Actinobacteriota families (F) in colonic and fecal samples on PD29.

β-Diversity of colonic and fecal microbiome were presented as Jaccard similarity network (Figure 1A) and Bray–Curtis distance in a PCoA plot (Figure 1B). The Jaccard model considers only the presence or absence of taxa, whereas the Bray–Curtis model accounts for both presence/absence and relative abundances of taxa. The Jaccard plot highlighted distinct microbial compositions in both inulin-supplemented groups (HIP and HIS) compared with those in AI and HI. The biggest cluster consisted of all AI samples and several colon and fecal samples from HI, suggesting substantial overlap in taxa profiles of these samples. However, a small cluster consisting of only HI samples underscored a unique change of taxa caused by high-iron intake. The Bray–Curtis dissimilarity plot showed significant differences in all pairwise comparisons (*P* < 0.001) except HIP compared with HIS in colon and feces (*P* ≥ 0.5) and AI compared with HI in feces (*P* = 0.12). In comparison between sample types within treatment, significant difference between colonic and fecal β-diversity was only detected in the AI group (*P* = 0.04). Inulin supplementation (HIP and HIS) had major impact on β-diversity in both colonic and fecal microbiome, primarily differentiating from AI and HI on axis 1 (25.64%), whereas iron excess had a moderate effect that was partly reflected by the marginal separation from AI samples on axis 2 (10.07%).

The PCoA biplot showed top 5 microbial taxa that drove separation of colonic sample clusters, with the length and direction of the taxon vectors indicating the relative contribution to the observed separation (Figure 1C). The analysis revealed significant enrichment of *Bacteroides* (genus) in AI and HI, whereas *Lachnospiraceae NK3A20 group* (genus) and Selenomonadaceae family are the most distinct taxon features characterizing inulin-supplemented groups (HIP and HIS).

### Relative abundance of colonic and fecal microbial taxa on PD29

On PD29, Firmicutes and Bacteroidetes dominated colonic and fecal microbiome across treatment groups, accounting for over 94% of the total composition. Actinobacteriota was the next most abundant phylum, with relative abundance higher in HIP (2.6% in colon and 4.9% in feces) compared with the other groups (Table 2). Other phyla—including Proteobacteria, Desulfobacterota, Euryarchaeota, Fusobacteriota (data not shown), and Synergistota (data not shown)*—*collectively constituted <2% or 4% of the colonic and fecal microbiome, respectively. Dietary treatments influenced colonic Firmicutes (*P* = 0.052) and Bacteroidetes (*P* = 0.056) abundance, with Firmicutes being more abundant in HIS, whereas AI and HI had higher Bacteroidetes abundance. In feces, Bacteroidota abundance was greater in HI (23.7%) than that in HIP (11.5%; *P* < 0.05), whereas Firmicutes was lower in HI (71.7%) than that in HIS (83.6%; *P* < 0.05).

**TABLE 2:** Relative abundance (%) of bacterial phyla in colon and feces on postnatal day 29.

Bacterial phylum	Site	AI	HI	HIP	HIS
Actinobacteriota	Colon	0.91	0.53	2.59	0.99
	Feces	1.54	1.21	4.93	2.78
Bacteroidota	Colon	35.12^1^	35.81	25.57^1^	23.07
	Feces	14.21^ab^	23.72^a^	11.52^b^	13.12^ab^
Desulfobacterota	Colon	0.62^a^	0.3^ab^	0.09^c^	0.14^bc^
	Feces	1.39^a^	0.85^ab^	0.28^b^	0.31^b^
Euryarchaeota	Colon	0.13	0.24	0.01	0.01
	Feces	1.1	1.69	0.48	0.06
Firmicutes	Colon	61.92^1^	62.77	71.35	75.37
	Feces	80.28^ab^	71.71^b^	82.52^ab^	83.55^a^
Proteobacteria	Colon	0.25	0.18	0.35	0.12
	Feces	0.13	0.07	0.21	0.11

Values are presented as mean.

Abbreviations: AI, iron-adequate milk diet; HI, high-iron milk diet; HIP, high-iron milk diet with prebiotic; HIS, high-iron milk diet with symbiotic.

^a,b^Within the site, values that share no common letter differed significantly across treatments.

1 Within the treatment, values differed significantly between sites.

Within colonic Firmicutes, 7 families were significantly affected by treatment (*P* ≤ 0.02) (Figure 1D). AI was associated with the highest abundance of Christensenellaceae and Oscillospiraceae, with significant higher concentrations than that in HIP and HIS (*P* < 0.05). Similarly, Leuconostocaceae was more abundant in AI than those in in HIP and HIS (*P* < 0.05). In both colon and feces, AI and HI had greater Ruminococcaceae but lower Selenomonadaceae and Veillonellaceae than those in HIP and HIS (*P* < 0.001). Fecal Leuconostocaceae was more abundant in AI than that in HIS (*P* < 0.05). Although not statistically significant, Lactobacillaceae abundance was consistently higher in colon than that in feces, whereas the opposite trend was observed for Ruminococcaceae.

Among colonic Bacteroidetes families, AI and HI had higher Bacteroidaceae and Rikenellaceae but lower Prevotellaceae abundances than HIP and HIS (*P* < 0.01) (Figure 1E). Similar treatment effects were observed in fecal samples, although differences were less pronounced and less statistical significance in pairwise comparisons. Compared with fecal samples, Bacteroidaceae was more abundant in colon in AI and HI groups but lower in HIP and HIS group. Prevotellaceae was consistently more abundant in colon than in feces across treatments.

For Actinobacteriota families, Atopobiaceae exhibited large individual variation and did not differ significantly between treatment in either colon or feces (Figure 1F). Bifidobacteriaceae remained at low concentrations (<0.1%) across treatment groups.

At the genus level, the 10 most abundant genera were presented in Table 3. In colon, *Bacteroides* abundance was significantly higher in AI and HI than that in HIP and HIS. In feces, it was more abundant in HI than that in HIP and HIS and in AI than that in HIS (*P* < 0.05). *Colidextribacter* was more abundant in both colon and feces in AI and HI than in HIP and HIS, with significant differences (*P* < 0.05) in AI compared with HIP/HIS, HI compared with HIS in colon, and HI compared with HIS in feces. In both colon and feces, *Ruminococcus* was more abundant in AI and HI than that in HIP and HIS. In contrast, *Megasphaera*, *Lachnospiraceae_NK3A20_group*, and *Prevotella* were more abundant in HIP and HIS compared to AI and HI (*P* < 0.001), except for fecal *Prevotella*, where the difference between HIP and HI was not significant. *Lactobacillus* was more abundant in the colons of HI than that in AI (*P* < 0.05), but its abundance in feces was not affected by treatment. *Mitsuokella* was most abundant in HIS, with significantly higher concentrations than AI and HI in the colon and AI in feces (*P* < 0.05). Comparing colonic and fecal samples, *Lactobacillus* and *Prevotella* were generally more abundant in the colon, whereas *Ruminococcus* was more prevalent in feces.

**TABLE 3:** Relative abundance (%) of the most abundant bacterial genera in colon and feces on postnatal day 29.

Bacterial genus	Site	AI	HI	HIP	HIS
*Bacteroides*	Colon	19.02^a,^^1^	23.67^a^	1.05^b,^^1^	1.66^b^
	Feces	5.63^ab^	16.55^a^	3.44^bc^	2.53^c^
*Blautia*	Colon	5.16	4.47	6.37	3.89
	Feces	4.20	5.39	6.47	6.75
*Colidextribacter*	Colon	5.27^a^	2.49^ab^	1.02^bc^	0.20^c^
	Feces	2.73^ab^	4.58^a^	1.34^ab^	0.92^b^
*Holdemanella*	Colon	4.34	2.38	4.63	4.96
	Feces	2.88	3.18	3.64	6.00
*Lachnospiraceae_NK3A20_group*	Colon	0.04^b^	0.06^b^	8.20^a^	7.78^a^
	Feces	0.08^b^	0.07^b^	8.67^a^	7.08^a^
*Lactobacillus*	Colon	6.61^b^	17.27^a,^^1^	9.09^ab,^^1^	6.84^ab^
	Feces	2.07	3.92	2.02	1.78
*Megasphaera*	Colon	0.52^b^	1.52^b^	10.17^a^	12.40^a^
	Feces	0.86^b^	0.92^b^	6.90^a^	9.37^a^
*Mitsuokella*	Colon	0.09^c^	1.96^bc^	2.38^ab^	8.46^a^
	Feces	0.09^b^	1.07^ab^	2.99^a^	6.85^a^
*Prevotella*	Colon	5.68^b,^^1^	1.62^b^	18.07^a,^^1^	15.77^a,^^1^
	Feces	1.00^b^	0.16^b^	1.67^ab^	4.07^a^
*Ruminococcus*	Colon	4.47^a,^^1^	5.06^a^	0.29^b^	0.11^b^
	Feces	11.85^a^	10.10^a^	0.61^b^	0.67^b^

Abbreviations: AI, iron-adequate milk diet; HI, high-iron milk diet; HIP, high-iron milk diet with prebiotic; HIS, high-iron milk diet with synbiotic.

^a,b,c^Within the site, values that share no common letter differed significantly across treatments.

1 Within the treatment, the abundance differed significantly between sites.

### Diversity of fecal microbiome

The 16S rRNA sequencing yielded an mean of 38,940 reads per sample, which reduced to 10,869 per sample after the quality filtering, removal nontarget reads, and rarefaction processes. The phyloseq object of fecal samples identified 1301 unique taxa. The Shannon index showed no significant treatment effect (*P* = 0.46) but was significantly affected by day (*P* < 0.001) and treatment × day interaction (*P* = 0.01) (Table 4). Similarly, the Chao1 index was affected by the interaction (*P* < 0.001), with higher values in AI than that in HIS on PD8 (P= 0.03), in HI than that in HIP on PD15 (*P* = 0.09), and in AI than that in HIS on PD29 (*P* = 0.06) (Table 4). A trend was observed for higher Chao1 index in AI and HI than that in HIP and HIS (*P* ≤ 0.06), whereas Chao1 index continuously increased in all groups throughout the study (*P* < 0.001).

**TABLE 4:** α-Diversity of fecal microbiomes.

Index	PD	AI	HI	HIP	HIS
Chao1	2	175.1 ± 6.4^x^	142.1 ± 11.8^x^	166.3 ± 16.3^x^	174.1 ± 12.4^y^
	8	214.9 ± 6.0^ay^	200.4 ± 16.4^aby^	178.4 ± 17.5^bcxy^	153.8 ± 15.0^cx^
	15	225.7 ± 6.7^yz^	227.5 ± 10.3^yz^	202.8 ± 14.1^xyz^	205.9 ± 12.5^yz^
	22	237.3 ± 7.5^yz^	233.9 ± 8.3^z^	221.9 ± 10.3^z^	217.2 ± 8.4^z^
	29	245.7 ± 8.0^z^	237.1 ± 10.2^z^	232.8 ± 12.6^z^	221.5 ± 12.1^z^
Shannon	2	3.4 ± 0.1^x^	3.0 ± 0.3^x^	3.5 ± 0.1	3.5 ± 0.1
	8	3.7 ± 0.1^xy^	3.8 ± 0.1^y^	3.6 ± 0.2	3.6 ± 0.1
	15	3.7 ± 0.1^xy^	3.9 ± 0.1^y^	3.6 ± 0.1	3.6 ± 0.1
	22	3.8 ± 0.1^y^	3.8 ± 0.1^y^	3.8 ± 0.1	3.7 ± 0.1
	29	4.0 ± 0.1^y^	3.7 ± 0.1^xy^	3.9 ± 0.1	3.8 ± 0.1

Values are presented as mean ± SE.

Abbreviations: AI, iron-adequate milk diet; HI, high-iron milk diet; HIP, high-iron milk diet with prebiotic; HIS, high-iron milk diet with symbiotic; PD, postnatal day.

^a,b,c^Within the day, values that share no common letter differed significantly across treatments.

^x,y,z^Within the treatment, values that share no common letter differed significantly across days.

**FIGURE 2:**
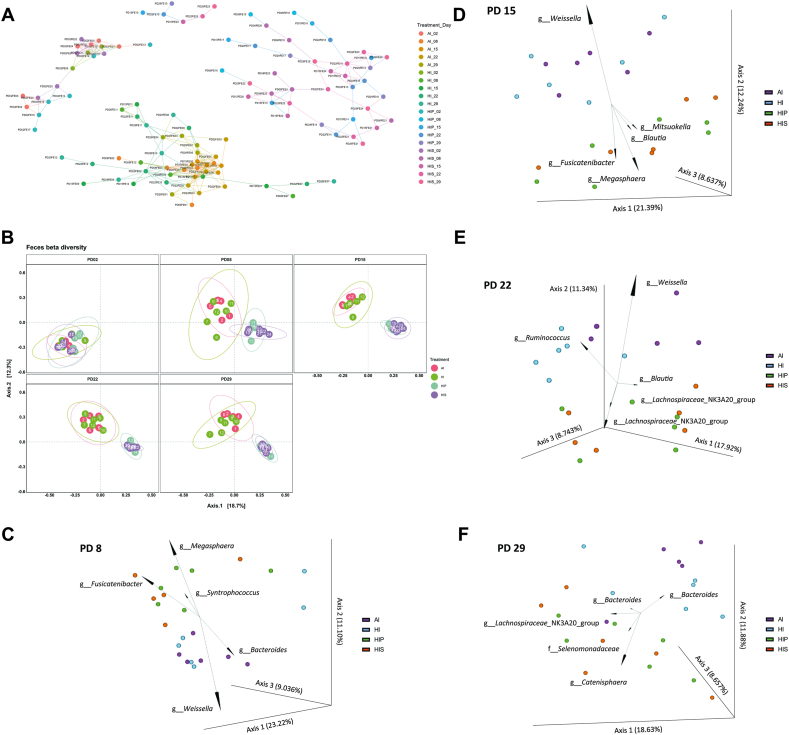
β**-**Diversity and relative abundance of fecal microbiota. The β-diversity of fecal microbiome presented as Jaccard network plot (A) and Bray–Curtis distance in a principal coordinates analysis (PCoA) plot (B). PCoA biplots highlighted key genera contributing to sample clustering on postnatal day (PD)8 (C), PD15 (D), PD22 (E), and PD29 (F).

The Jaccard network revealed 4 main clusters (Figure 2A): *1*) a cluster containing all PD2 baseline samples from all treatments (left), *2*) a large cluster comprising all AI and HI samples except those from PD2 (bottom), and *3*) 2 distinct clusters representing HIP and HIS samples (top and right). This clustering pattern indicates significant shifts in taxonomic profiles over time compared with baseline and highlights the impact of inulin supplementation. Despite the overall clustering of nonbaseline AI and HI samples, there were fewer connections between most HI and AI samples, suggesting limited overlap in their taxonomic profiles. Similarly, Bray–Curtis dissimilarity showed that HIP and HIS samples formed distinct clusters, separate from AI and HI on axis 1 since PD8 (*P* < 0.01) (Figure 2B). AI samples were marginally separated from HI along axis 2, with significant dissimilarity confirmed on PD15, PD22, and PD29 (*P* < 0.01). Overall, inulin significantly influenced fecal microbiome diversity, dietary iron had a moderate effect, and *L agilis* YZ050 did not exhibit additional influence on diversity.

The PCoA biplots identified the top 5 taxa that influenced the sample clustering on PD8, PD15, PD22, and PD29 (Figure 2C–F). On PD8, inulin-fed pigs had higher *Megasphaera*, *Fusicatenibacter*, and *Syntrophococcus* species, whereas AI and HI groups were enriched in *Bacteroides* and *Weissella* species (Figure 2C). On PD15, *Megasphaera* and *Fusicatenibacter* species remained the prominent taxonomic features of HIP and HIS, followed by *Blautia* and *Mitsuokella* species. *Weissella* species was the key distinguishing taxon for AI and HI (Figure 2D). By PD22, HIP and HIS samples were characterized by greater *Blautia* and *Lachnospiraceae NK3A20 group* species, whereas AI and HI samples were marked by *Weissella* and *Ruminococcus* species, respectively (Figure 2E). On PD29, *Lachnospiraceae NK3A20 group*, *Catenisphaera* species, and Selenomonadaceae were enriched in inulin-fed pigs, whereas *Bacteroides* became the primary feature in AI and HI (Figure 2F).

### Relative abundance of fecal microbial taxa

**FIGURE 3:**
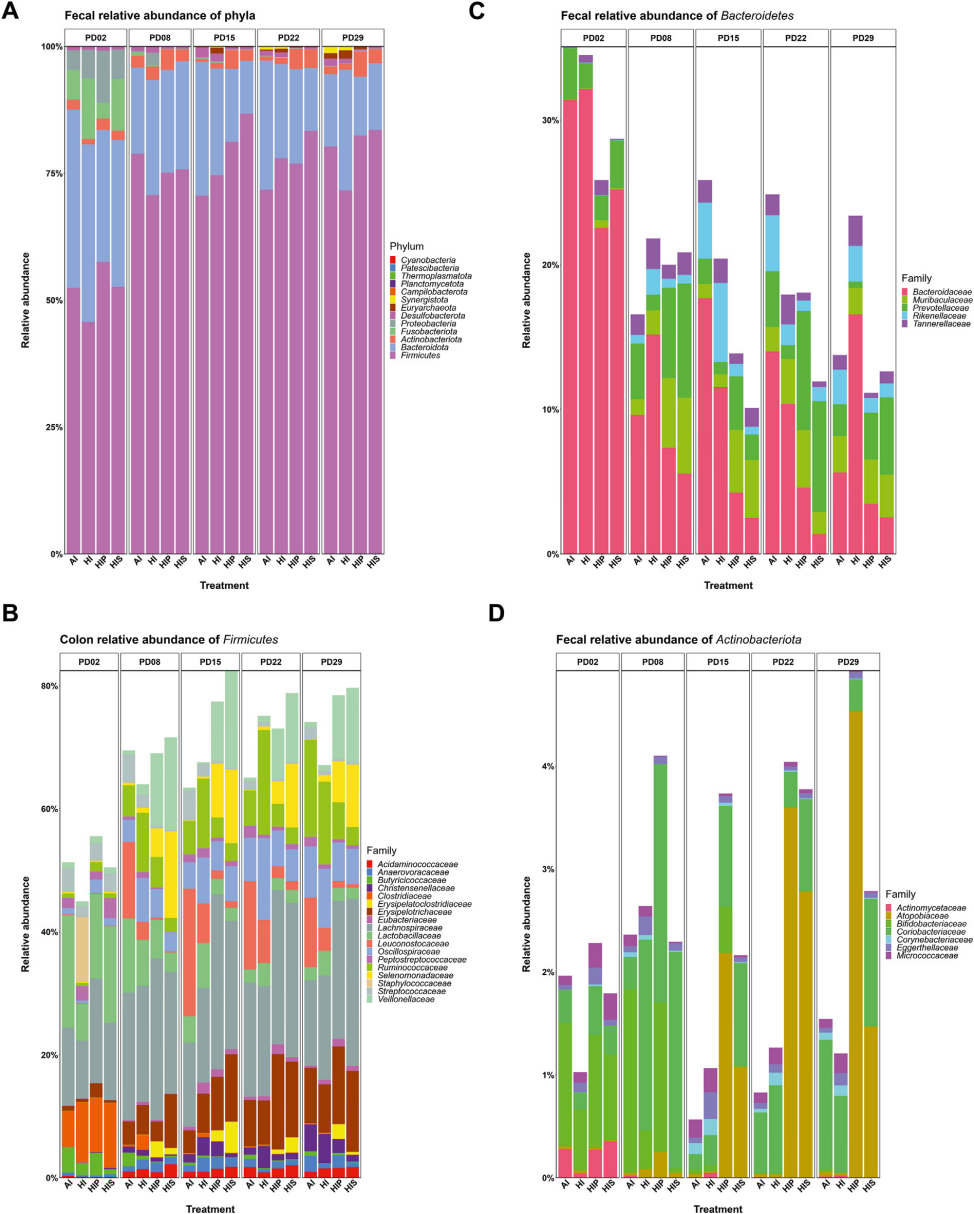
Relative abundance of fecal microbiome. The stacked bar graphs presented relative abundance of main phyla (A) and families of Firmicutes (B), Bacteroidetes (C), Actinobacteriota (D) in fecal microbiome on postnatal day (PD)2 (baseline), PD8, PD15, PD22, and PD29.

Mirroring the colon microbiota, fecal microbiota was dominated by Firmicutes (70.2%–86.4%), Bacteroidetes (10.6%–26.8%), and Actinobacteriota (0.6%–5.2%) across treatments and days (Figure 3A, Table 5). Other phyla remained below 1%. Kruskal–Wallis rank sum test showed significant treatment×day interactions for Bacteroidetes, Firmicutes, Desulfobacterota, Euryarchaeota, Fusobacteriota, Proteobacteria, and Synergistota (*P* < 0.001). Proteobacteria abundance was higher in the HIS (PD8, 15) and HIP (PD15) than in AI and HI (*P* ≤ 0.012).

**TABLE 5:** Relative abundance (%) of bacterial phyla in feces.

Bacterial phylum	PD	AI	HI	HIP	HIS
Actinobacteriota	8	2.36	2.64	4.10	2.29
	15	0.57^b^	1.06^ab^	3.73^a^	2.16^a^
	22	0.83	1.27	4.04	3.77
	29	1.54	1.21	4.93	2.78
Bacteroidota	8	16.94	22.62	20.23	21.30
	15	26.33^a^	20.98^ab^	14.37^ab^	10.37^b^
	22	25.44	18.53	18.63	12.36
	29	14.21	23.72	11.52	13.12
Desulfobacterota	8	0.89	1.15	0.43	0.44
	15	1.90^a^	1.49^a^	0.41^b^	0.46^b^
	22	0.97^a^	0.91^a^	0.15^b^	0.27^b^
	29	1.39^a^	0.85^ab^	0.28^b^	0.31^b^
Euryarchaeota	8	0.02^x^	0.07^x^	0.00^x^	0.00^x^
	15	0.12^y^	1.14^y^	0.20^y^	0.10^y^
	22	0.21^y^	0.79^y^	0.16^y^	0.09^y^
	29	1.10^z^	1.69^y^	0.48^y^	0.06^y^
Firmicutes	8	78.89	70.75	75.14	75.81
	15	70.60^b^	74.66^ab^	81.22^ab^	86.79^a^
	22	71.76	77.98	76.89	83.35
	29	80.28	71.71	82.52	83.55
Proteobacteria	8	0.25	2.44	0.05	0.04
	15	0.32^a^	0.26^a^	0.03^b^	0.04^b^
	22	0.10	0.06	0.06	0.04
	29	0.13	0.07	0.21	0.11

Values are presented as mean.

Abbreviations: AI, iron-adequate milk diet; HI, high-iron milk diet; HIP, high-iron milk diet with prebiotic; HIS, high-iron milk diet with symbiotic; PD, postnatal day.

^a,b,c^Within the day, values that share no common letter differed significantly across treatments.

^x,y,z^Within the treatment, values that share no common letter differed significantly across days.

Among families of Firmicutes, Lactobacillaceae, Leuconostocaceae, Ruminococcaceae, Selenomonadaceae, and Veillonellaceae were significantly affected by either main effect(s) or interactions (*P* < 0.05) (Figure 3B). Although Lactobacillaceae was affected by an interaction, post-hoc analysis found only trends (PD8: AI > HIS, P= 0.08; PD15: HI > HIS, P= 0.09). Leuconostocaceae was most abundant in AI, followed by HI, and lowest in HIP and HIS (*P* < 0.05). Ruminococcaceae abundance was highest in HI, followed by AI and HIP, and lowest in HIS, with varied differences across days. Selenomonadaceae and Veillonellaceae were more abundant in HIS and HIP than in AI and HI throughout the study (*P* < 0.001).

Among Bacteroidetes, all families were significantly affected by treatment×day interaction (*P* ≤ 0.03) (Figure 3C). Bacteroidaceae was higher in AI than HIP and HIS on PD15, 22 (*P* < 0.01), and marginally on PD29 (*P* < 0.1). Additionally, HI had higher abundance than HIS on PD15 (*P* < 0.05) and higher than HIP and HIS on PD 22, 29 (*P* < 0.05). Prevotellaceae was more abundant in HIP than HI on PD15, 29 (*P* < 0.05). The abundance of Rikenellaceae was higher in AI and HI than in HIP and HIS on PD15 (*P* < 0.05). By PD 22, AI had the highest Rikenellaceae abundance (*P* < 0.001).

Actinobacteriota families showed considerable variation. Bifidobacteriaceae was rare in piglet’s gut (<1.8% on PD 2, 8; <0.5% thereafter) (Figure 3D). Atopobiaceae was more abundant in HIP than AI and HI on PD15, 22, and 29.

At genus level, significant treatment×day interaction effect (*P* < 0.03) was observed on most genera analyzed in the study, including more abundant genera *Bacteroides, Colidextribacter*, *Lachnospiraceae NK3A20 group*, *Lactobacillus*, *Megasphaera, Mitsuokella*, *Prevotella*, and *Ruminococcus* (Table 6). *Bacteroides* was higher in AI than HIP and HIS, and higher in HI than HIS on PD15, 22 (*P* < 0.05), and more abundant in HI than HIP and HIS on PD29 (*P* < 0.05). *Lactobacillus* (*P* < 0.1) and *Lachnoclostridium* were the highest in the HI group compared to the other groups, whereas both HIP and HIS had lower *Lachnoclostridium* than AI and HI (*P* < 0.05). *Catenisphaera* (data not shown), *Fusicatenibacter* (data not shown), *Megasphaera* and *Mitsuokella* were also enriched in inulin-fed groups throughout the study (*P* < 0.01), except for *Mitsuokella* on PD29, where no differences were found. Similarly, *Prevotella* was more abundant in inulin-fed groups from PD15–29 (*P* < 0.1). Conversely, the abundance of *Ruminococcus* was lower in inulin-fed groups than in AI since PD8 (*P* < 0.05), whereas HI was associated with the highest *Ruminococcus*.

**TABLE 6:** Relative abundance (%) of the most abundant bacterial genera in feces.

Bacterial genus	PD	AI	HI	HIP	HIS
*Bacteroides*	8	9.60	15.16	7.34	5.55
	15	17.68^a^	11.54^a^	4.25^b^	2.48^b^
	22	13.98^a^	10.35^a^	4.58^b^	1.38^c^
	29	5.63^ab^	16.55^a^	3.44^bc^	2.53^c^
*Blautia*	8	7.99	5.44	5.08	7.87
	15	4.60	1.53	3.18	8.93
	22	7.05	7.76	8.41	7.21
	29	4.20	5.39	6.47	6.75
*Colidextribacter*	8	0.29	0.56	0.34	0.17
	15	0.74^b^	1.79^a^	0.92^b^	0.91^b^
	22	0.87	7.03	1.27	1.15
	29	2.73^ab^	4.58^a^	1.34^ab^	0.92^b^
*Holdemanella*	8	2.10	2.73	2.29	4.02
	15	1.81	1.66	3.39	5.56
	22	5.50	4.46	7.29	8.27
	29	2.88	3.18	3.64	6.00
*Lachnospiraceae_NK3A20_group*	8	0.07	0.05	0.07	0.06
	15	0.04	0.07	6.83	0.08
	22	0.06	0.06	4.00	10.95
	29	0.08^b^	0.07^b^	8.67^a^	7.08^a^
*Lactobacillus*	8	12.04	7.45	6.26	3.17
	15	4.12	7.32	2.50	2.05
	22	2.02	3.74	1.88	2.06
	29	2.07	3.92	2.02	1.78
*Megasphaera*	8	0.66^b^	1.46^b^	10.51^a^	11.22^a^
	15	0.36^b^	0.31^b^	7.87^a^	11.65^a^
	22	0.58^b^	0.99^b^	6.19^a^	7.50^a^
	29	0.86^b^	0.92^b^	6.90^a^	9.37^a^
*Mitsuokella*	8	0.38^b^	0.78^b^	3.47^a^	11.55^a^
	15	0.05^b^	0.31^b^	6.46^a^	8.49^a^
	22	0.10^c^	0.61^c^	2.16^b^	9.39^a^
	29	0.09^b^	1.07^ab^	2.99^a^	6.85^a^
*Prevotella*	8	1.56	0.21	4.89	5.84
	15	0.38	0.52	2.23	1.08
	22	2.04^ab^	0.14^b^	5.83^a^	6.49^a^
	29	1.00^b^	0.16^b^	1.67^ab^	4.07^a^
*Ruminococcus*	8	2.60^a^	6.31^a^	0.25^b^	0.42^b^
	15	1.98^b^	5.90^a^	0.21^c^	0.28^c^
	22	3.02^a^	11.70^a^	0.42^b^	0.37^b^
	29	11.85^a^	10.10^a^	0.61^b^	0.67^b^

Values are presented as mean.

Abbreviations: AI, iron-adequate milk diet; HI, high-iron milk diet; HIP, high-iron milk diet with prebiotic; HIS, high-iron milk diet with symbiotic; PD, postnatal day.

^a,b,c^Within the day, values that share no common letter differed significantly across treatments.

## Discussion

This preclinical study used a milk-fed piglet model to simulate formula-fed infants. Our previous findings demonstrated that the HI milk markedly increased hepatic iron deposition as well as colonic and fecal iron concentrations compared with AI milk. However, inulin supplementation, regardless of the presence of *L.* *agilis* YZ050, reduced hepatic iron accumulation and lowered colonic and fecal iron concentrations induced by HI milk [5]. This companion study investigated the effects of excess dietary iron on gut microbiome and explored the role of inulin, along and in combination with inulin-fermenting *L.* *agilis*, in modulating gut microbiota under condition of abundant iron fortification in milk formula.

### Microbiotic effects of iron fortification

Most infant formulas in the United States contain 12 mg Fe/L, a concentration considerably higher than that in breastmilk. A recent study using the stable iron isotopes showed that fractional iron absorption from such a formula was only 3.2% in early infancy [33], highlighting substantial unabsorbed iron reaching the colon and influencing microbiota. Several studies have reported adverse effects of iron fortification or supplementation on gut microbiota of infants and young children in resource-poor settings [9,10,15,34,35]. These effects include reduced abundance of beneficial commensals, particularly *Bifidobacterium* and *Lactobacillus* species, increased colonization by pathogenic bacteria, heightened intestinal inflammation, and increased diarrheal incidence. Conversely, in healthy, nonanemic Swedish infants, consuming a high-iron–fortified formula (6.6 mg Fe/d) did not increase pathogenic bacteria in stool, although *Bifidobacterium* species abundance declined [36]. In our pig model, fecal consistency was scored twice daily, revealing occasional mild diarrhea across all treatment groups. However, HI-fed pigs had the least days of diarrhea [5]. Nevertheless, all pigs were apparently healthy and free of severe diarrheal illness during the study. It should be noted that the study was conducted in a specific pathogen-free facility with daily cleaning of feeding apparatuses, and the milk replacer provided adequate nutrition to support growth. The baseline health status and environmental factors, such as hygiene and pathogen exposure, may influence the outcome of iron fortification on gut microbiota and risk of enteric infections.

The phylogenetic diversity of gut microbiota is enriched in early infancy in both humans and pigs [37,38]. Similarly, in this study, fecal α-diversity increased from baseline (PD2) across all treatments. However, compared with AI pigs, colonic α-diversity (Shannon and Chao1 indices) decreased in all groups fed the HI milk, but such a difference was not observed in fecal α-diversity on PD29. Similar reductions in colonic or cecal microbial diversity have been reported in 3-wk-old weaned pigs and suckling rats following dietary iron fortification or oral iron supplementation [8,39]. In contrast, clinical trials that analyzed fecal microbial diversity of infants usually found no impact of iron fortification [10,35]. In this study, the colon–fecal discrepancies regarding the response to iron fortification extended to β-diversity and composition profile of certain taxa (discussed further). Feces are usually the only available samples for analysis of gut microbiome in clinical trials. However, our findings suggested that fecal microbiome may not comprehensively represent the impact of iron on gut microbiota at primary fermentation site.

*Bifidobacterium* and *Lactobacillus* species play critical roles in infant gut health by preventing pathogen colonization. Iron availability was shown to modulate these commensals. Bifidobacterial strains isolated from iron-deficient infants exhibit varying capacities for siderophore production and iron uptake [40]. When cocultured with enteropathogens under low-iron conditions, strains with high-iron sequestration significantly inhibited *Salmonella typhimurium* and/or enterohemorrhagic *E. coli* growth [41]. Unlike most bacteria, *Lactobacillus* does not require iron for growth [6]. Depleting iron with chelators enhanced *Lactobacillus* and *Bifidobacterium* species growth in in vitro fermentation of children fecal microbiota compared with the fermentation under normal iron condition; such an effect was not observed under low-iron or high-iron conditions [42].

Studies in breastfed Kenyan infants and Ivorian schoolage children found that iron-fortified MNPs or biscuits favored enterobacteria growth over lactobacilli and/or bifidobacteria [9,10], which potentially contributed to heightened gut inflammation and diarrheal illness. High prevalence of anemia (67.3%), iron deficiency (25.5%), and enteric pathogens was detected at baseline in the study with Kenyan infants [10]. Similarly, oral iron supplementation significantly reduced *Lactobacillus* and *Bifidobacterium* species in cecal contents of suckling rats compared with those in controls [8]. However, in a study of 6-mo-old Swedish infants who were healthy and iron replete, high-iron formula (6.6 mg Fe/d) increased fecal *Lactobacillus* species relative abundance without enhancing pathogenic bacteria compared with the group fed the low-iron formula (1.2 mg Fe/d), despite reduced *Bifidobacterium* species [36].

Suckling piglets naturally have a much lower *Bifidobacterium*—<1% of total bacterial taxa—compared with human infants, where it is the dominant genus [43]. In our study, HI milk did not alter *Bifidobacterium* relative abundance (< 0.1%) in colonic and fecal samples. Similar to findings in Swedish infants, *Lactobacillus* abundance in colon was higher in HI than that in AI. A similar study with artificially reared piglets found that *Lactobacillus* abundance in the ascending colon was significantly higher in those receiving iron-deficient milk (2.7 mg Fe/L) compared with that in those receiving control-iron milk (21.3 mg Fe/L) [44]. Overall, it seems iron supplementation or fortification may decrease *Lactobacillus* and *Bifidobacterium* when subjects are iron deficient at baseline. However, many studies, including ours, reported only relative abundance, which reflects compositional shifts rather than absolute changes. Future research should incorporate absolute quantification of gut commensals to better understand the impact of iron fortification.

### Microbiotic effects of inulin and symbiotic

In contrast to the marginal effects of iron fortification, inulin supplementation significantly altered both the diversity and composition of the gut microbiome. Previous studies have shown that prebiotic supplementation, such as GOS, can mitigate the unfavorable effects of iron fortification on the gut microbiome [15]. Long-chain inulin is a prebiotic fiber known for its bifidogenic and *Lactobacillus* species–enhancing properties [17,18]. We initially hypothesized that inulin supplementation would promote restoration of these beneficial commensals whose growth can be adversely affected by iron fortification. However, unexpectedly, inulin supplementation did not increase the relative abundance of these microbes. Instead, broader microbiota shifts were observed as early as PD8, particularly within the phyla Firmicutes and Bacteroidota.

Within Firmicutes, the most significant enrichments were in the families Lachnospiraceae, Veillonellaceae, and Selenomonadaceae, with the primary affected genera being *Lachnospiraceae NK3A20 group*, *Megasphaera*, and *Mitsuokella*, respectively. These genera have been previously identified as inulin-responsive bacteria capable of binding and using inulin in human gut [45]. Consistently, these genera also emerged as signature taxa in colonic and fecal samples distinguishing inulin-fed piglets, as confirmed by the PCoA biplot in our study. *Lachnospiraceae NK3A20 group* and *Megasphaera* species, both abundant in the rumen, express a set of glycosyl hydrolases—enzymes required for breaking down glycosidic bonds in complex carbohydrates [46,47]. However, in vitro study have shown that *Lachnospiraceae NK3A20* does not grow when cultured with 0.1% inulin alone, suggesting that it may function as a secondary metabolizer of oligosaccharides, relying on other fibrolytic bacteria to initiate the breakdown of plant polysaccharides [46]. Most species within in these genera are short-chain fatty acid (SCFA) producers, particularly butyrate, which serves as an energy substrate for colonocytes and confers benefits to gut health and intestinal barrier functions [46,48]. Notably, Dou et al. [49] identified Lachnospiraceae as one of the signature taxa characterizing the gut microbiome of healthy piglets. Supporting our findings, dietary supplementation of 10% long-chain inulin from chicory roots has been shown to increase fecal *Megasphaera* species abundance in 4-wk-old weaned pigs [50].

Inulin supplementation also significantly affected the Bacteroidota phylum, shifting the dominant genus from *Bacteroides* (observed in AI and HI piglets) to *Prevotella* in colon. Similar findings have been reported in 4-wk-old weaned pigs fed inulin-supplemented diets at either 5% or 10% (wt/wt), which exhibited increased *Prevotella* species abundance alongside a reduction in *Bacteroides* species [50,51]. The features of *Bacteroides* or *Prevotella* species dominance have been used to define 2 distinct enterotypes of gut microbiome in human adults [52]. This enterotype concept has recently been extended to the gut microbiota of infants. The developmental transition from *Firmicutes* and *Bifidobacterium* species dominance to a microbiota enriched with *Bacteroides* or *Prevotella* communities has been observed in the second and third year of life, denoting a normal maturation process toward adult-like microbiome [53]. The *Prevotella* enterotype is characterized by a robust fiber-fermenting capacity and high SCFA production, especially propionate, whereas the *Bacteroides* enterotype is more efficient at degrading and using animal proteins and fats compared with plant fibers [54]. Both gestational factors (e.g. gestational weight gain and delivery mode) and postnatal dietary influences (e.g. breastfeeding compared with formula feeding) play critical roles in shaping gut microbiota and establishment of enterotype in early life [55]. Once established, enterotypes appeared to be more resistant to dietary interventions in adulthood as observed in some studies [56,57]. Notably, in overweight or obese adults, a high *Prevotella*-to-*Bacteroides* ratio has been linked to greater weight loss in response to a high-fiber diet [58]. Nevertheless, few research has examined the long-term metabolic and health implications of different enterotypes established in early infancy and childhood.

*Lactobacillus agilis* YZ050, a strain isolated from milk-fed calves, exhibits strong extracellular β-fructosidase activity, enabling it to efficiently catabolize long-chain inulin and release fructose. We hypothesized that cosupplementation of inulin with *L agilis* YZ050 would produce a synbiotic effect, facilitating inulin fermentation and crossfeeding other lactic acid bacteria [25]. However, no significant differences in microbial diversity or taxonomic composition were observed between the HIS and HIP groups, suggesting minimal synergistic effect. A follow-up qPCR analysis of fecal DNA samples revealed only moderate colonization of *L agilis* YZ050 in HIS piglets [5], which may explain its limited impact on gut microbiota. Additionally, synbiotic supplementation did not alter colonic or fecal pH, further indicating its minor role in modulating enteric fermentation. Given that *L agilis* YZ050 is not a native bacterium in pig’s digestive tract [5], optimizing dosing frequency should be considered in future research.

### Discrepancies between colon and fecal microbial responses

Gut microbiota is influenced by various luminal factors, including pH, oxygen availability, nutrient composition, and chemical exposure. Consequently, microbial diversity and composition fluctuate substantially across different intestinal segments. A study examining spatial variations in the gut microbiome of 7-wk-old weaned pigs demonstrated that microbial diversity (α-diversity) is much higher in all anatomical sites of large intestine (cecum, colon, and rectum) than that in the small intestine and feces [59]. Similarly, β-diversity revealed considerable heterogeneity in microbiota composition across intestinal sites and fecal samples. Although the fecal microbiome more closely resembles that of the large intestine than the stomach or small intestine, notable differences exist at all taxonomic levels. Specifically, fecal samples consist of significantly higher relative abundance of *Firmicutes* and *Actinobacteria* species and a lower abundance of *Bacteroidetes* species than colonic samples. These phylum-level compositional differences between colonic and fecal microbiomes were also observed in the current and a previous study on younger, milk-fed piglets [44]. However, neither study on milk-fed piglets detected significant difference in α-diversity between colonic and fecal microbiomes [44], suggesting that diet may play a crucial role in shaping species diversity. Furthermore, our findings indicate that not all effects of iron fortification and inulin supplementation on colonic microbiome persisted in the fecal microbiome. For example, Chao1 index in colonic microbiome was significantly reduced by iron fortification but was not affected in feces. Additionally, the relative abundance of *Lactobacillus* species was significantly higher in the HI than that in the AI group in the colon but not in feces. Inulin supplementation also significantly altered the abundance of *Bacteroides* and *Prevotella* in the colon compared with both AI and HI, whereas these differences between HIP and HI and/or AI groups diminished in feces. Although fecal microbiome is commonly used as a proxy for gut microbiome, our study highlights potential discrepancies of fecal microbiome in representing the exact effects of dietary interventions on gut microbiome.

In conclusion, this preclinical study shows that excess iron fortification in milk formula had minor impact on gut microbiome and did not adversely affect key beneficial commensals, such as *Bifidobacterium* and *Lactobacillus* species. However, contextual factors such as hygiene, baseline iron status, and pathogen exposure should be considered when interpreting these findings or comparing them with clinical trials that evaluated iron fortification at infancy in resource-poor settings.

Despite the lack of effect on bifidobacteria and lactobacilli, inulin supplementation markedly altered microbial diversity and composition in milk-fed piglets. Inulin enriched *Firmicutes* families associated with fiber fermentation and SCFA production. It also shifted the dominant Bacteroidetes genus from *Bacteroides* to *Prevotella*. In contrast, synbiotic supplementation with *L.* *agilis* YZ050 had minimal effects, likely due to limited colonization. These findings highlight inulin’s role in shaping early-life gut microbiota and emphasize the need for further research on its long-term health implications.

## Author contributions

The authors’ responsibilities were as follows – PJ: designed the research and obtained funding support; BLL: contributed to research conceptualization; JP: conducted the research and sample and data analysis; CJ, YL: contributed to method development and data analysis; SW, DAM: provided probiotic inoculants; JP, PJ: wrote the manuscript; and all authors: read and approved the final manuscript.

## Data availability

All data are reported in the article.

## Funding

This study was supported by the USDA-NIFA Hatch Multistate Research Fund, Rustici Seed Grant from Department of Nutrition, and Novo Nordisk Foundation (Grant No.: NNFSA210073688).

## Conflict of interest

The authors report no conflicts of interest.
